# Flowing Liquid Crystal Torons Around Obstacles

**DOI:** 10.3390/mi15111302

**Published:** 2024-10-26

**Authors:** Júlio P. A. Santos, Mahmoud Sedahmed, Rodrigo C. V. Coelho, Margarida M. Telo da Gama

**Affiliations:** 1 Centro de Física Teórica e Computacional, Faculdade de Ciências, Universidade de Lisboa, 1749-016 Lisboa, Portugal; 2 Departamento de Física, Faculdade de Ciências, Universidade de Lisboa, 1749-016 Lisboa, Portugal; 3 Independent Researcher, Cairo 11528, Egypt; 4 International Institute for Sustainability with Knotted Chiral Meta Matter, Hiroshima University, Higashihiroshima 739-8511, Japan

**Keywords:** liquid crystals, topology, hydrodynamics, lattice Boltzmann method

## Abstract

Liquid crystal torons, localized topological structures, are known for their stability and dynamic behaviour in response to external stimuli, making them attractive for advanced material applications. In this study, we investigate the flow of torons in chiral nematic liquid crystals around obstacles. We simulate the fluid flow and director field interactions using a hybrid numerical method combining lattice Boltzmann and finite difference techniques. Our results reveal that the toron dynamical behaviour depends strongly on the impact parameter from the obstacle. At impact parameters smaller than half cholesteric pitch, the flowing toron is destabilized by the interaction with the obstacle; otherwise, the flowing toron follows a trajectory with a deflection which decays exponentially with the impact parameter. Additionally, we explore the scattering of torons by multiple obstacles, providing insights into how the dynamics of these structures respond to complex environments.

## 1. Introduction

Liquid crystals (LCs) represent a fascinating class of soft materials characterized by their fluidity combined with anisotropic properties, making them highly responsive to external fields and boundary conditions. This unique combination has led to widespread applications, particularly in display technologies, and has also spurred interest in fundamental research on the stability of their phases and topological structures [1]. Among these structures, solitons in chiral nematic LCs have emerged as a significant area of study due to their rich topological properties and potential for novel technological applications [2,3]. Besides skyrmions, which are two-dimensional configurations, many other topological-protected structures were realized in experiments, including torons, hopfions, and solitonic macromolecules. We will refer to torons as the three-dimensional elementary skyrmion terminating at two-point defects to satisfy the uniform surface boundary conditions and match the topologically nontrivial skyrmion tube with the uniform far-field background and reserve skyrmion to describe the two-dimensional mid-plane structure.

Solitons in liquid crystals are localized, non-singular distortions of the director field that exhibit robust particle-like behaviour and complex interactions. These structures are of particular interest due to their ability to be manipulated by external stimuli, such as electric fields, which can drive their motion and induce collective behaviour [4,5]. The study of soliton dynamics in LCs has provided insights into fundamental aspects of topology in soft matter and has highlighted their potential in advanced material applications, including reconfigurable optical devices and microfluidic systems [4,6,7,8,9,10,11,12,13].

While much of the research on LC torons has focused on their behaviour under uniform external fields, little is known under non-uniform conditions such as static colloids or other obstacles trapped in the LC matrix. Furthermore, only a handful of studies have addressed the interaction between torons and mass flows [14,15,16,17], which has thus remained an overlooked problem of relevance in various fields. Understanding the dynamical response of LC torons in non-uniform or heterogeneous media is relevant for practical applications, where flowing torons may encounter various types of obstacles, such as geometrically patterned substrates, dispersed colloidal particles or defects in the LC matrix. This response will significantly alter the torons’s initial trajectory, in addition to its shape and stability, potentially driving novel dynamical phenomena.

In the theoretical study of torons, three approaches are commonly used in the literature. The first approach involves the numerical solution of continuum equations resulting from the minimization of the elastic free energy, which is typically implemented using finite difference or finite volume methods [4,18,19,20]. This approach has focused either on the dynamic evolution of torons or on the stable configuration of static torons. The second approach is based on analytical studies of the continuum equations, under simplified conditions required to make the problems tractable [21,22]. This method often provides valuable insights into the toron behaviour in idealized situations. The third approach, a more recent development, uses particle-based modelling, where torons are treated as individual particles [23]. Langevin dynamics is employed to simulate their motion, and potential functions for the toron interactions are inferred from experimental data or from simulations using the first method. Moreover, there are attempts to use neural networks to measure system parameters using optical images obtained from experiments on skyrmions [24]. In this study, we use the first approach, which is combined with the lattice Boltzmann method, to simulate material flow, providing a comprehensive and versatile framework to study the interaction between topology and external mass flows that may be perturbed by an arbitrary array of obstacles.

In what follows, we study the flow of liquid crystal torons, driven by an external mass flow, around solid obstacles. The toron’s stability and trajectory are found to depend strongly on the impact parameter of the obstacle. When the impact parameter is less than h<5μm, the toron becomes unstable and disintegrates, and flows where torons would otherwise remain stable. However, when the impact parameter is larger than h≥5μm, the stability of the toron is preserved while the toron’s trajectory is deflected by an amount that decays exponentially with the impact parameter. Notably, the toron’s velocity is found to increase as it approaches the obstacles due to flow constriction. We have also examined the dynamics and stability of torons flowing around one and between two obstacles, providing additional insights into how multiple obstacles may influence the dynamics of flowing torons.

## 2. Method

### 2.1. Hydrodinamic Equations

The dynamics of the liquid crystal (LC) director field are described by the Ericksen–Leslie model [25,26,27,28]. This model couples two dynamical equations: one for the material flow and another for the director field. These equations are particularly adequate to describe the behaviour of LCs in the nematic or cholesteric phases.

For the velocity field, we use the Navier–Stokes equation along with the continuity equation [29]:

(1) $$\begin{array}{ccc} & & \rho \partial_{t}u_{\alpha }+\rho u_{\beta }\partial_{\beta }u_{\alpha }=\partial_{\beta }\left[-p\delta_{\alpha \beta }+\sigma_{\alpha \beta }^{v}+\sigma_{\alpha \beta }^{e}\right]+\rho g \end{array}$$

(2) $$\begin{array}{ccc} & & \partial_{\alpha }u_{\alpha }=0, \end{array}$$

where the viscous stress tensor is defined as

(3) $$\begin{array}{cc} \sigma_{\alpha \beta }^{v}= & \alpha_{1}n_{\alpha }n_{\beta }n_{\mu }n_{\rho }D_{\mu \rho }+\alpha_{2}n_{\beta }N_{\alpha }+\alpha_{3}n_{\alpha }N_{\beta }\\ & +\alpha_{4}D_{\alpha \beta }+\alpha_{5}n_{\beta }n_{\mu }D_{\mu \alpha }+\alpha_{6}n_{\alpha }n_{\mu }D_{\mu \beta }. \end{array}$$

In these equations, ρ denotes the fluid density, *g* is the external acceleration that drives the fluid flow, *p* is the pressure, u is the fluid velocity, n is the director field describing the direction of alignment of the LC molecules, and $\alpha_{n}$ is the Leslie viscosity. The kinematic transport, which describes the influence of the macroscopic flow field on the microscopic structure, is given by

(4) $$\begin{array}{c} N_{\beta }=\partial_{t}n_{\beta }+u_{\gamma }\partial_{\gamma }n_{\beta }-W_{\beta \gamma }n_{\gamma }, \end{array}$$

while the shear rate and vorticity tensors are defined as

(5) $$\begin{array}{c} D_{\alpha \mu }=\frac{1}{2}\left(\partial_{\alpha }u_{\mu }+\partial_{\mu }u_{\alpha }\right),\;W_{\alpha \mu }=\frac{1}{2}\left(\partial_{\mu }u_{\alpha }-\partial_{\alpha }u_{\mu }\right). \end{array}$$

The elastic stress tensor is

(6) $$\begin{array}{c} \sigma_{\alpha \beta }^{e}=-\partial_{\alpha }n_{\gamma }\frac{\delta E}{\delta (\partial_{\beta }n_{\gamma })}, \end{array}$$

where *E* represents the Frank–Oseen elastic free energy:

(7) $$\begin{array}{c} E=\int dV\left(\frac{K_{11}}{2}{\left(\nabla \cdot n\right)}^{2}+\frac{K_{22}}{2}{\left(n\cdot \left[\nabla \times n\right]+q_{0}\right)}^{2}+\frac{K_{33}}{2}{\left[n\times \left[\nabla \times n\right]\right]}^{2}\right). \end{array}$$

where $K_{11}$, $K_{22}$ and $K_{33}$ are the Frank elastic constants, and $q_{0}=2\pi /P$, with as *P* the cholesteric pitch.

The second set of equations describes the evolution of the director field:

(8) $$\begin{array}{cc} \; & \partial_{t}n_{\mu }=\frac{1}{\gamma_{1}}h_{\mu }-\frac{\gamma_{2}}{\gamma_{1}}n_{\alpha }D_{\alpha \mu }-u_{\gamma }\partial_{\gamma }n_{\mu }+W_{\mu \gamma }n_{\gamma }, \end{array}$$

where $\gamma_{1}=\alpha_{3}-\alpha_{2}$ is the rotational viscosity determining the relaxation rate of the director, and $\gamma_{2}=\alpha_{3}+\alpha_{2}$ is the torsion coefficient characterizing the contribution to the viscous torque from the velocity field gradients. The ratio $\gamma_{2}/\gamma_{1}$ is known as the alignment parameter, with $|\gamma_{2}/\gamma_{1}|\;>\;1$ indicating flow-aligning and $|\gamma_{2}/\gamma_{1}|\;<\;1$ flow-tumbling systems. The molecular field is given by

(9) $$\begin{array}{c} h_{\mu }=-\frac{\delta E}{\delta n_{\mu }}. \end{array}$$

The simulations employed a hybrid numerical method. The velocity field is resolved using the lattice Boltzmann method [30,31], summarized in the next subsection, with the elastic and viscous stress tensors (except the term proportional to $\alpha_{4}$) introduced as force terms. The director field equation, Equation (8), was solved using a predictor–corrector finite difference algorithm [32]. The derivatives were calculated using central moment differences:

(10) $$\begin{array}{c} \frac{dF}{dx}=\frac{F(x+\Delta x)-F(x-\Delta x)}{2\Delta x}+O\left(\Delta x^{2}\right), \end{array}$$

where F is a generic function and Δx is the step. Note that the gradients are calculated only in the fluid nodes while keeping the quantities in the solid nodes constant. On solid boundaries, including the obstacles, infinite homeotropic anchoring and no-slip conditions were applied using the bounce-back condition [30].

The simulations started with the liquid at rest, and the directors mostly aligned perpendicular to the plates except near the toron, whose configuration was obtained by minimizing its free energy starting from the Ansatz of Ref. [14]. The material parameters were chosen to be close to those of MBBA at 22 °C [33], except for the absolute viscosity (or equivalently, $\alpha_{4}$), which was doubled to ensure reasonable simulation times while keeping its value of the same order of magnitude of the material one. Note that the physical behaviour is mainly controlled by the Ericksen number [14]: $Er\equiv \frac{\mu v_{t}P}{K}$, where $v_{t}$ is the toron velocity, *P* is the cholesteric pitch, μ is the viscosity, and *K* is the average elastic constant. If we multiply μ by two, $v_{t}$ must be divided by two in order to have the same Ericksen number and, thus, the same behaviour. The code was parallelized in CUDA-C, and the simulations were executed on GPUs [34]. A typical performance of the simulation is ∼328 MLUPS (Mega Lattice Updates Per Seconds) in a Nvidia Tesla V100 using double precision calculations.

The use of GPUs allowed us to perform 3D simulations in a feasible time frame. By comparison, our previous C++ implementation, parallelized with OpenMP and running on 8 CPU cores, was 2 orders of magnitude slower. Additionally, we found that the current method, based on the Ericksen–Leslie theory, is more suitable for simulating flowing liquid crystal torons than the Beris–Edwards model (as in Ref. [16]). It uses less memory (storing only the 3 components of the director field instead of the 6 components of the Q-tensor) and assumes a constant scalar order parameter, reducing spurious effects such as non-uniform far fields.

### 2.2. Lattice Boltzmann Method

We obtain the velocity field by solving the discretized Lattice Boltzmann Equation (LBE) using the single relaxation time (SRT) collision approximation and Guo’s forcing scheme [35], as follows:

(11) $$\begin{array}{cc} \; & f_{i}\left(x+e_{i}\Delta t,t+\Delta t\right)-f_{i}\left(x,t\right)=\\ & -\frac{\Delta t}{\tau }\left(f_{i}\left(x,t\right)-f_{i}^{\mathrm{eq}}\left(x,t\right)\right)+\Delta t\left(1-\frac{1}{2\tau }\right)S_{i}^{F}\left(x,t\right), \end{array}$$

where $f_{i}$ is the particle distribution function, and $f_{i}^{\mathrm{eq}}$ is the equilibrium distribution function, which is determined by

(12) $$\begin{array}{c} f_{i}^{\mathrm{eq}}\left(x\right)=w_{i}\rho \left(x\right)\left[1+\frac{e_{i}\cdot u\left(x\right)}{c_{s}^{2}}+\frac{{\left(e_{i}\cdot u\left(x\right)\right)}^{2}}{2c_{s}^{4}}-\frac{u(x)\cdot u(x)}{2c_{s}^{2}}\right], \end{array}$$

where $c_{s}$ is the lattice speed of sound, defined as $c_{s}=\frac{\Delta x}{\sqrt{3}\Delta t}$. Δx, Δt, and the reference density $\rho_{0}$ are set to unity (lattice units). $w_{i}$ and $e_{i}$ are the lattice weights and the discrete velocity vector in the $i^{\mathrm{th}}$ direction, respectively. In this work, we use the $D_{3}Q_{19}$ lattice arrangement; hence, $w_{i}$ and $e_{i}$ are given by

(13) $$e_{i}=\left(\begin{array}{ccccccccccccccccccc} 0 & 1 & -1 & 0 & 0 & 0 & 0 & 1 & -1 & 1 & -1 & 1 & -1 & 1 & -1 & 0 & 0 & 0 & 0\\ 0 & 0 & 0 & 1 & -1 & 0 & 0 & 1 & 1 & -1 & -1 & 0 & 0 & 0 & 0 & 1 & -1 & 1 & -1\\ 0 & 0 & 0 & 0 & 0 & 1 & -1 & 0 & 0 & 0 & 0 & 1 & 1 & -1 & -1 & 1 & 1 & -1 & -1 \end{array}\right),$$

(14) $$w_{i}=\left(\begin{array}{ccccccccccccccccccc} \frac{1}{3} & \frac{1}{18} & \frac{1}{18} & \frac{1}{18} & \frac{1}{18} & \frac{1}{18} & \frac{1}{18} & \frac{1}{36} & \frac{1}{36} & \frac{1}{36} & \frac{1}{36} & \frac{1}{36} & \frac{1}{36} & \frac{1}{36} & \frac{1}{36} & \frac{1}{36} & \frac{1}{36} & \frac{1}{36} & \frac{1}{36} \end{array}\right).$$

The macroscopic fluid density is defined as

(15) $$\rho \left(x\right)=\sum_{i}f_{i}\left(x\right).$$

The relaxation time (τ) is related to the kinematic viscosity of the fluid (ν) through

(16) $$\nu =c_{s}^{2}\left(\tau +\frac{1}{2}\right)\Delta t.$$

The forcing term in the LBE equation $S_{i}^{F}$ is defined as

(17) $$\begin{array}{c} S_{i}^{F}\left(x\right)=w_{i}\left[\frac{e_{i}\cdot F\left(x\right)}{c_{s}^{2}}+\frac{\left(e_{i}e_{i}-c_{s}^{2}I\right):\left(u\left(x\right)F\left(x\right)+F\left(x\right)u\left(x\right)\right)}{2c_{s}^{4}}\right], \end{array}$$

where F represents the resultant force acting on the flow field (external force and stresses arising from the liquid crystal distortions), and u is the macroscopic fluid velocity, which is defined as

(18) $$u\left(x\right)=\frac{1}{\rho (x)}\sum_{i}e_{i}f_{i}\left(x\right)+\frac{\Delta t}{2\rho (x)}F\left(x\right).$$

The bounce-back boundary condition is used to model the no-slip boundary condition over the solid nodes [30]. The idea behind the bounce-back boundary condition is to reflect the distribution functions hitting a solid node with the same magnitude in the reverse direction. The exact location of the solid boundary is assumed to be between two lattice points [30].

## 3. Results

We report the results of numerical simulations of a flowing toron driven by an external force, around a cylindrical pillar near its initial trajectory. The liquid crystal was initialized at rest, with alignment along the vertical direction, except at the toron, where we used the relaxed configuration obtained from the Ansatz [14]. The system dimensions were set to $L_{x}\times L_{y}\times L_{z}=96\times 56\times 16$, and the material parameters are listed in Table 1. The pillar, with a radius of R=3.13μm (or 5 Δx), homeotropic anchoring and no-slip boundary, was placed at $x=L_{x}/2$ and $y=L_{y}/2+h$. The fluid was driven by an external body force of magnitude g=312.5 m/s^2^ (or $2\times 10^{-9}$ l.u.) in the *x* direction.

**Figure 1:**
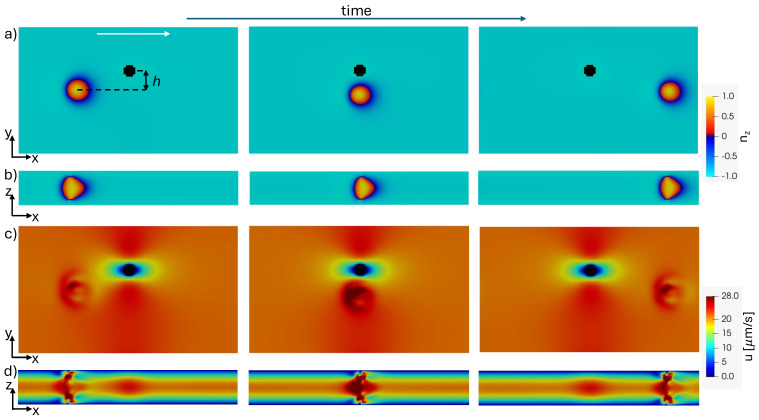
Snapshots of a toron flowing close to a pillar: (**a**) z-component of the director field in the mid-xy plane; (**b**) z-component of the director field in the xz plane that intercepts the geometric centre of the toron; (**c**) magnitude of the velocity field in the mid-xy plane; and (**d**) the magnitude of the velocity field in the xz plane that intercepts the geometric centre of the toron. The white arrow indicates the direction of the flow. The impact parameter of the obstacle is h=5μm.

As expected, the pillar distorts the velocity field and perturbs the toron initial trajectory, as shown in Figure 1. In the mid-plane, Figure 1a, the toron shrinks slightly as it flows around the pillar but later recovers its original configuration. To confirm the toron’s stability post-pillar interaction, we relaxed the final configuration in Figure 1 without the external force, confirming that it remains stable (not shown). The toron cross-section in Figure 1b reveals that the toron becomes asymmetric in the direction of the flow, consistent with previous observations in Poiseuille-like flows without the pillar [16]. The velocity field is perturbed by the toron, while the pillar introduces large velocity gradients in the flow field (Figure 1c). In the cross-sectional view, Figure 1d, the flow exhibits a Poiseuille-like profile, except near the toron and the pillar.

**Figure 2:**
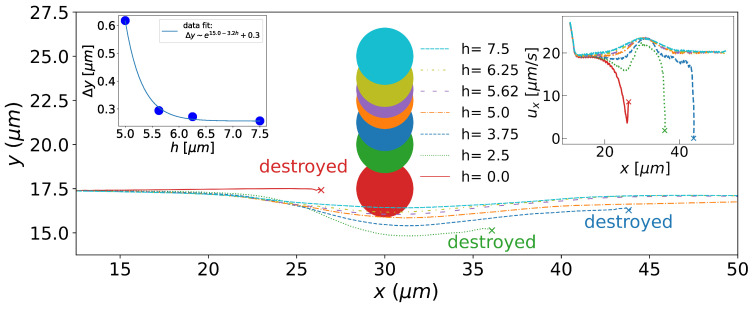
Trajectories of torons deflected by pillars at different impact parameters, i.e., the lateral distance between the toron’s initial trajectory and the pillar’s centre, *h* (legend in μm). The colour of the pillars is the same as that of the corresponding trajectories. Torons that are destabilized and destroyed by the impact are indicated in the figure. The inset on the left depicts the dependence of the vertical deflection of the toron trajectory, Δy, on the impact parameter, *h*. The solid line is an exponential fit to the data. The inset on the right shows the toron velocity as a function of the position.

**Figure 3:**
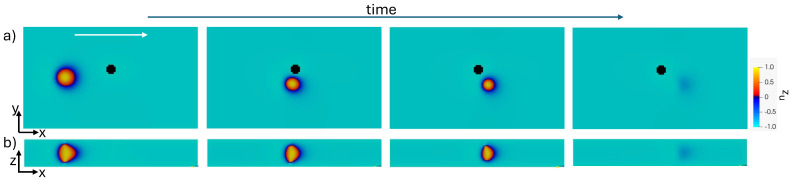
Unstable toron flowing around a pillar: (**a**) z-component of the director field in the xy mid-plane; and (**b**) z-component of the director field in the xz plane that follows the geometric centre of the toron. The white arrow indicates the direction of the flow. The impact parameter of the obstacle is h=2.5μm.

The deflection of the initial trajectory of the toron depends on the impact parameter *h*, as shown in Figure 2. Trajectories are deflected significantly when the impact parameter is less than the cholesteric pitch. However, below a threshold, h=3.75 μm or 0.43 P, in our simulations, the toron becomes unstable and disintegrates. The toron in Poiseuille flows already becomes unstable without the obstacle at higher speeds [17], and here, it occurs due to the local increase in the velocity field around the obstacle. When the toron collides directly with the pillar (h=0), it disintegrates before flowing around the obstacle. For small but non-zero values of *h*, the toron flows around the pillar but later becomes unstable, as depicted in Figure 3.

The inset on the left of Figure 2 depicts the deflection Δy of the toron initial trajectory as a function of the impact parameter, *h*. As *h* increases, Δy decays exponentially due to elastic distortions of the director field. Exponential decays are usually observed around defects or distortions in liquid crystal textures [36]. However, it is clear that the decay reported here does not tend to zero as *h* tends to infinity, as expected for elastic distortions only. This is likely due to the finite size of the flow domain in the *y* direction. The constriction in the velocity field, caused by the obstacle at $x=L_{x}/2$, leads to a velocity increase in this region, which in turn leads to a deflection. The inset on the right shows the toron velocity as a function of position, where we note that a stable toron accelerates near the pillar, with its velocity increasing by about 26% before returning to its initial value, due to the flow constriction by the obstacle. Notice that this increase in the velocity field occurs even at the largest value of *h*.

**Figure 4:**
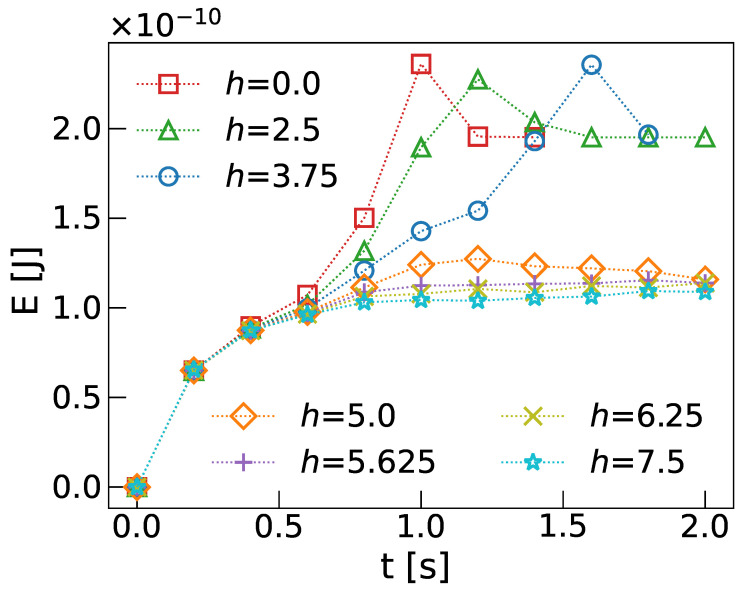
Time evolution of the free energy of flowing torons around pillars, for different impact parameters *h* in μm.

The free energy integrated over the entire volume, calculated using Equation (7), is plotted in Figure 4 as a function of time for various impact parameters, *h*. At small *h*, the free energy rises sharply, reaching a peak when the toron loses stability and disintegrates. At larger impact parameters, *h*, the toron remains stable, and the free energy stabilizes, with slight perturbations occurring as the toron flows around the pillar, particularly evident in the orange curve (h=5μm) around t=1 s.

**Figure 5:**
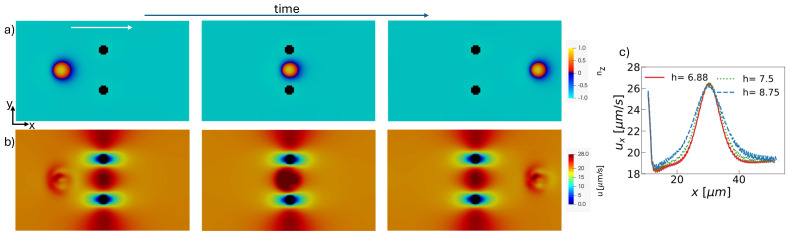
Snapshots in the mid-plane of a toron flowing through two obstacles. (**a**) Z-component of the director field. (**b**) Magnitude of the velocity field. The white arrow indicates the direction of the applied force. Each pillar has an impact parameter h=6.875 μm. (**c**) Toron velocity as a function of the position for different values of *h* in μm. The vertical lines on the top are the times when the toron flows between the pillars.

Until now, we considered the flow of a toron around a single pillar. To explore the effects of multiple obstacles, we simulated the toron flow between two pillars, which act as a flow constriction (see Figure 5). In this configuration, the toron accelerates by 44% as it flows between the pillars, as shown in Figure 5c. Similar to the single-pillar case, the toron becomes unstable at small values of *h*. In our simulations, the toron remains stable for h>6.25 μm, which is almost twice the impact parameter required for toron stability in the single-pillar case. This is likely due to the toron’s size reduction when passing around the pillars, an effect that is amplified by the presence of two obstacles. Additionally, in this configuration, the toron does not deviate from its initial trajectory.

In addition to simulating the interaction between the toron and a cylindrical pillar with homeotropic anchoring, we investigated other obstacle geometries (not shown). For example, we replaced the pillar with a solid sphere, mimicking a colloid, and applied different anchoring conditions (both homeotropic and toron-like). However, no qualitative differences were observed. Thus, we conclude that the primary factor determining the flow of torons around obstacles, as well as their stability, appears to be the effect of the large velocity gradients introduced by the obstacles rather than their detailed form.

## 4. Conclusions

In this study, we investigated the interaction between flowing liquid crystal torons and solid obstacles. Through numerical simulations using a combination of the lattice Boltzmann method and finite differences, we demonstrated how the dynamics of flowing torons respond to the presence of obstacles in the flow domain. Our findings reveal that the toron’s stability and trajectory depend strongly on the impact parameter of the obstacle. At short impact parameters (smaller than half cholesteric pitch), the toron becomes unstable and disintegrates, while at larger impact parameters, the toron’s trajectory is deflected around the obstacle. The deflection of the trajectory and the increase in velocity as the toron approaches the obstacle depend strongly on the impact parameter. In addition, we explored the dynamics of torons flowing between two obstacles, providing insights into how multiple obstructions influence the toron´s stability and motion. An extension of this work could address the study of flowing torons in a lattice or in disordered arrays of obstacles. Moreover, it is also interesting to investigate the dynamics of flowing torons in gradient velocity fields. Symmetry breaking resulting in deflections of the toron trajectories in directions perpendicular to the drive, as reported in Couette-like flows [15], may be a general phenomenon that is also observed here. An extension of this work could involve the study of flowing torons in a lattice of obstacles and in a disordered distribution of them. Moreover, it would be interesting to investigate the dynamics of torons in gradients of velocity field in the mid-plane. Possibly, they may break the symmetry and move to transverse directions like in Couette-like flows [15].

This study contributes to our understanding of toron interactions in complex environments which could be used in applications in reconfigurable optical devices and microfluidic systems.

## Figures and Tables

**Table 1:** Parameters used in the simulation and physical units.

Symbol	Simulation Units	Physical Units
ρ	1	1088 Kg/m^3^
Δx	1	0.625 μm
Δt	1	2$\;\times \;10^{-9}$ s
$K_{11}$	$1.67\;\times \;10^{-7}$	$6.4\;\times \;10^{-12}$ N
$K_{22}$	$7.88\;\times \;10^{-8}$	$3.0\;\times \;10^{-12}$ N
$K_{33}$	$2.62\;\times \;10^{-7}$	$9.98\;\times \;10^{-12}$ N
$\alpha_{1}$	0.0373	0.0036 Pa·s
$\alpha_{2}$	−0.4496	−0.044 Pa·s
$\alpha_{3}$	−0.0203	−0.0020 Pa·s
$\alpha_{4}$	0.9318	0.091 Pa·s
$\alpha_{5}$	0.3084	0.030 Pa·s
$\alpha_{6}$	−0.1617	−0.016 Pa·s
*P*	14	8.75 μm
$L_{x}$, $L_{y}$, $L_{z}$	56, 56, 16	35, 35, 10 μm

## Data Availability

The data that support the findings of this study are available from the corresponding author, upon reasonable request.
